# Pedestrian Detection at Day/Night Time with Visible and FIR Cameras: A Comparison

**DOI:** 10.3390/s16060820

**Published:** 2016-06-04

**Authors:** Alejandro González, Zhijie Fang, Yainuvis Socarras, Joan Serrat, David Vázquez, Jiaolong Xu, Antonio M. López

**Affiliations:** 1 Autonomous University of Barcelona, Cerdanyola, Barcelona 08193, Spain; zfang@cvc.uab.es (Z.F.); ysocarras@cvc.uab.es (Y.S.); joans@cvc.uab.es (J.S.); dvazquez@cvc.uab.es (D.V.); jiaolong@cvc.uab.es (J.X.); antonio@cvc.uab.es (A.M.L.); 2 Computer Vision Center, Cerdanyola, Barcelona 08193, Spain

**Keywords:** far infrared, day/nighttime, pedestrian detection

## Abstract

Despite all the significant advances in pedestrian detection brought by computer vision for driving assistance, it is still a challenging problem. One reason is the extremely varying lighting conditions under which such a detector should operate, namely day and nighttime. Recent research has shown that the combination of visible and non-visible imaging modalities may increase detection accuracy, where the infrared spectrum plays a critical role. The goal of this paper is to assess the accuracy gain of different pedestrian models (holistic, part-based, patch-based) when training with images in the far infrared spectrum. Specifically, we want to compare detection accuracy on test images recorded at day and nighttime if trained (and tested) using (a) plain color images; (b) just infrared images; and (c) both of them. In order to obtain results for the last item, we propose an early fusion approach to combine features from both modalities. We base the evaluation on a new dataset that we have built for this purpose as well as on the publicly available KAIST multispectral dataset.

## 1. Introduction

Visual pedestrian detection has received attention for more than a decade from computer vision researchers due to its multiple applications in Advance Driver Assistance Systems (ADAS) [1,2,3], autonomous vehicles [4] and video surveillance [5,6,7], being nowadays still a challenging problem. The accuracy of pedestrian detection methods remains limited because of occlusions, cluttered backgrounds and, foremost, bad visibility because of the varying lighting conditions under which they must operate.

Most efforts on building pedestrian detectors have focused on two directions, each being a key component of the whole system. The first one is the design of the features on which the statistical classifiers will work. Since the breakthrough of histograms of oriented gradients (HOG) by Dalal *et al.* [8], many other features and combinations of features have been proposed in the last decade, like HOG plus local binary patterns (LBP) [9], HOG plus color self similarity (CSS) [10], Haar features plus histogram of edges [11], integral channels [12] or macrofeatures [13], just to name a few. These features are *arranged* to form models: holistic [8,9], part-based (e.g., the DPM) [14,15,16], or patch based [17,18] , many times taking into account also different views and resolutions [14,19,20]. Another recent trend has been to complement those appearance-based features, computed from single frames, with additional motion and depth features such as in [21,22,23,24,25].

The second main direction has been the design of the classifier itself. Since the plain binary max-margin discriminative classifiers were employed in the initial approaches, we now see a plethora of classification architectures like cascades of classifiers [26,27], random forests of local experts [17], and even alternative approaches like generative classifiers [28], active learning [29], and domain adaptation [30,31]. In the last three years, there has also been an explosion of end-to-end learning of object models based on deep convolutional neural networks (deep CNNs) [32]. These models are mainly operating in the visible spectrum to leverage object annotations from image classification datasets given the large number of annotated object examples these deep CNNs need to converge to a useful object model. The reason is the huge number of parameters to learn, on the order of millions.

In parallel to all these works, there is a relatively unexplored third direction, namely, image acquisition. Recent works have started to supplement or even replace images provided by monochrome and color cameras in the visible spectrum with images from other modalities, with the intent of improving the performance of the whole system but still keeping the same types of features and classifiers.

**Figure 1:**
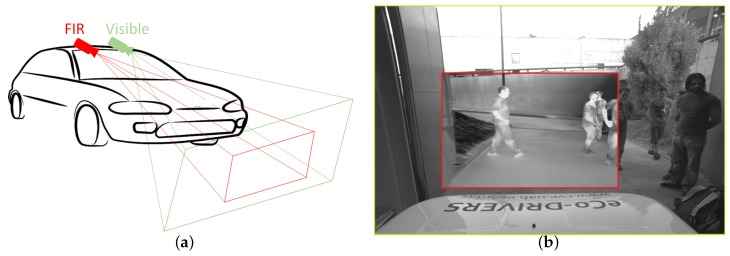
Camera setup for the CVC-14 dataset and registered sample frames showing the different field of views. (**a**) Fields of view of the visible and far infrared cameras and (**b**) example images.

**Figure 2:**
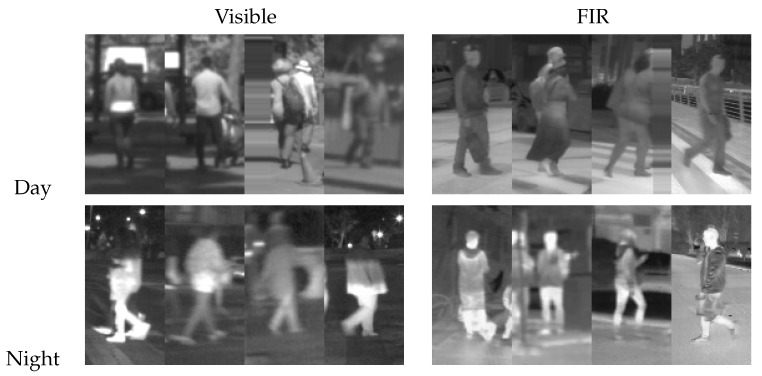
Sample pedestrians from the CVC-14 dataset.

Near infrared cameras, sensing in the range 0.75–1.3 μm, have been used for pedestrian detection in [33]. Far infrared cameras, instead, work in the range 7.5–13 μm. They have the distinctive advantages of leveraging the fact that the human body emits radiation around 9.3 μm [34] and their relative invariance to different illumination conditions (see Figure 1 and Figure 2), which may improve the detector robustness, as shown in [35,36,37,38,39,40].

The goal of this paper is to assess the accuracy of a pedestrian detector with regard to (1) the imaging modalities; (2) strong baselines in terms of features and pedestrian models proposed for this task; and (3) the lighting conditions. Even though we expect to get better results on sequences recorded at night with a far infrared (FIR) camera than with a standard color or monochrome camera, there are still relevant open questions in relation to the design of a practical and affordable pedestrian detector system. For instance, how does an FIR camera perform at daytime? Is its performance similar to that of a regular camera? Is it worth to combine features extracted from a color and an FIR camera operating simultaneously? If so, what is then the gain in accuracy at day and nighttime?

The contributions of this paper are:

- An extensive evaluation of pedestrian detectors for a number of combinations of the former three factors: visible/FIR modalities, pedestrian models and lighting conditions.
- We make available the new CVC-14 dataset in the Dataset section of http://adas.cvc.uab.es. CVC-14 is a new dataset of multimodal (FIR plus visible) videosequences and the corresponding detection groundtruth, comparable to the only other publicly available KAIST dataset [36].
- We assess the relevance of simultaneously using two cameras of different modality (FIR, Visible) by applying early fusion, which is done on KAIST.

In the following, we will review the works most related to ours and point out the main differences (Section 2). Section 3 presents our new dataset and compares it to KAIST. Based on both of them, we have designed and run a number of experiments, and present the results in Section 5. Finally, Section 6 summarizes this work and draws the conclusions.

## 2. Related Works

Recently, a number of works have appeared that explore the application of FIR cameras to pedestrian detection. This has probably been fostered by a drastic reduction of their price, which may favor its adoption by the automotive industry in the future. We divide the approaches into two categories.

The first one includes the approaches mainly focused on the introduction of new features, specifically targeted to this imaging modality. In this group, we find works like Olmeda *et al.* [41], which presents a new descriptor, the histograms of oriented phase energy (HOPE) and an adaptation of the latent variable SVM approach to FIR images. HOPE is a contrast invariant descriptor that encodes a grid of local oriented histograms extracted from the phase congruency of the images computed from a joint of Gabor filters. Besbes *et al.* [39] propose a pipeline for pedestrian detection in FIR images using a hierarchical codebook of SURF in the head region, taking advantage of the brightness of this area inside the regions of interest (ROIs). Another nice work by Li *et al.* [38] employs sparse coding. Overall, these works try to show that FIR cameras and specialized features improve over standard cameras and “off-the-shelf” features previously employed in this and other domains. The problem is that, given the absence of benchmark datasets, it becomes difficult to do a fair quantitative comparison. For instance, the total number of pedestrians present in the sequences, number of occlusions, the distribution of the pedestrian distance to the camera (size in pixels), the type of background present, the frame resolution and the frame rate *etc*. are factors that clearly have an influence on the results.

In the second category, we consider those papers mainly addressing the evaluation and comparison of modalities and features, as we intend to do in this work. We have found just a few papers on this category, all of them published less than one year ago. Miron *et al.* [37] evaluate a set of different descriptors over visible and FIR sequences. This evaluation is performed in on-board sequences but recorded only in the daytime.

More interesting, Yuan *et al.* [36] are the first to perform a comprehensive study and make publicly available their dataset. They take as baselines pedestrian detectors based on the aggregated channel features (ACF) originally proposed by Dollar *et al.* [3], but adding several combinations of new gradient orientation-related features computed from the FIR image intensity, resulting in the combination of features of both modalities. However, differently from them, we perform an exhaustive experimental analysis to demonstrate the advantages in detection using different modalities in isolation for different state-of-the-art detectors during different time/illumination conditions. Then, these results are used to propose a multi-modal approach combining visible and FIR spectrum images. We are more interested in the performance of state-of-the-art features and classifiers for pedestrian detection, not in the introduction of new features. At most, we want to investigate the effect of combining in a simple way features from different modalities, in the event of visible and FIR sequences simultaneously recorded. Ultimately, we want to set a baseline for future research and also identify the source of the improvements if any: e.g., a given set of features, image modality, specific lighting conditions, *etc.*, and for each case being able to perform a quantitative evaluation. Note that it is important to have a quantitative evaluation on the use of two different types of cameras simultaneously, since car manufacturers would like to use a single camera for ADAS to reduce overall cost and aesthetic impact.

## 3. Datasets

To build our new dataset we use both visible and FIR cameras to gather two long pairs of video sequences of day and night activity, respectively (see Figure 1). One pair was recorded at daytime, the other at night. We used an IDS UI-3240CP (IDS Imaging Development Systems GmbH, Obersulm, Germany) and an FLIR Tau 2 camera (FLIR Systems, Nashua, NH, USA), with the specifications in Table 1.

Note that resolution and the field-of-view do not match. Hence, we needed to perform an automatic spatial alignment and crop. Even though the cameras are not at the same position, the baseline is small and, once registered, the disparity and occlusions of objects beyond a few meters are negligible.

Table 2 shows the number of frames and annotated pedestrian for each of the four sequences in the dataset (here called CVC-14): day/FIR, night/FIR, day/visible and night/visible. This dataset was acquired at 10 FPS. We have defined a threshold for the minimum height of pedestrians that we will take into account later in the experiments. That is, we have annotated all of them but, as it is usually done [3], we will consider as mandatory the detection of those whose bounding box is higher than 50 pixels, about 10% of the registered frames height.

The KAIST multispectral pedestrian dataset [36] is a set of video sequences composed by 95 K frame pairs. The images from each pair have been recorded by an on-board color and thermal cameras at 20 Hz, both at a resolution of 640 × 480 pixels. Hence, it is well suited for pedestrian detection studies because the two underlying sequences, color and infrared, are synchronized. In addition, a beamsplitter in the acquisition setup makes each pair spatially registered so that the computed local features in both images correspond to the same region. Another important characteristic is the groundtruth with 103,128 dense annotations featuring people, cyclists and 1182 unique pedestrians.

## 4. Features and Classifiers

In this study, we have selected a short list with the most used and top scoring features and classifiers from the pedestrian detection literature. As for the features, they are HOG [8], LBP [9] and their aggregation as a single feature, which we will denote as HOG+LBP. As for the classifiers, we have selected three different types of models: holistic, learned by a linear SVM [42]; a patch-based classifier learned by a Random Forest of local experts [17]; and the popular DPM) [14]. Whereas the first one is probably well known for its wide application in many classification problems, we will shortly introduce the two latter, which may be more specific. The Random Forest of local experts (RF) [17] is a patch-based detector. RF is an ensemble of trees where each node is based on an SVM classifier learned on a random patch. In this way, different parts (patches) are selected to create a decision tree from which a classification score is computed on the basis of the probabilities of being a target object at the leaf node. The DPM [14] is a successful part-based detector that defines a fixed number of parts. Each of them are detected separately and a deformation cost is learned based on the part positions in the training samples. All of the part descriptions plus the deformation costs are concatenated to form a final descriptor, on which an SVM performs the final classification. The learning process is based on Latent SVM since the object parts are not supposed to be annotated, just the object as a whole is given.

We thus consider different combinations for the comparison: {HOG, LBP, HOG+LBP} × {Linear SVM, RF, DPM}. For each of them, we will assess the performance of the detector at day and night sequences separately. In addition, in each case, still, we will build a feature vector with the corresponding type of feature computed just on the visible frame, infrared frame, and the aggregation of both of these feature vectors.

In order to check whether complementarity information is better for pedestrian detection, we explore the integration of the two image modalities, visible and FIR. We thus propose to use an approach similar to [25]: for each candidate window, we extract HOG and LBP features over each modality and then combine them into a single feature vector to feed the classifier. We combine the features using an early fusion approach whereby the resulting descriptor is the plain concatenation of the features from each modality. It is worth mentioning also that, although for stereo-based systems it is possible to use a scene-based generation of candidate windows to be classified [1,2], in this paper, the visible and FIR modalities are treated as monocular systems regarding candidate generation. Thus, we use the scanning approaches defined in their respective works, which basically are the pyramidal sliding window [9,26] and the same for DPM but considering the detection of object parts at double the resolution of the whole object [14].

## 5. Experiments

### 5.1. Evaluation Protocol

As evaluation methodology, we follow the de-facto Caltech standard for pedestrian detection [3], *i.e.*, we plot curves of false positives per image (FPPI) *vs.* miss rate. The average miss rate (AMR) in the range of $10^{-2}$ to $10^{0}$ FPPI is taken as indicative of each detector accuracy, *i.e.*, the lower the better.

### 5.2. Experiments on the CVC-14 Dataset

**Figure 3:**
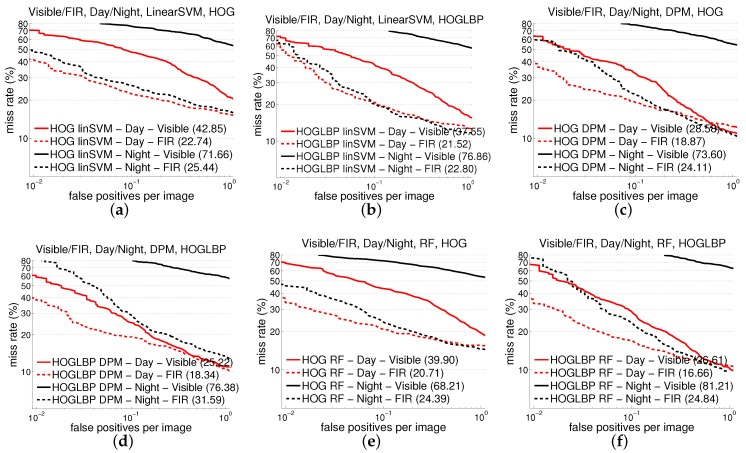
Results using different detectors over CVC-14 dataset. First row plot results using detectors based on (**a**) SVM/HOG, (**b**) SVM/HOG+LBP, (**c**) DPM/HOG, (**d**) DPM/HOG+LBP, (**e**) RF/HOG and (**f**) RF/HOG+LBP.

Table 3 summarizes the results in terms of AMR for the seven combinations of features and classifiers on the two lighting conditions and image modalities considered. For the holistic model (linear SVM), we test all of the features. For the patch-based one (RF of local experts), we keep HOG as reference, but the next test consists in directly combining HOG and LBP, since, for this model, we know from our previous work [17] (visible spectrum) that this combination works better than the two features in isolation. On the other hand, the standard (publicly available) DPM is based only in HOG, so following the same criterion as for RF, we have added LBP too, but it is not necessary to consider LBP alone. Overall, Table 3 plots the experiments that make more sense. We can appreciate that, as expected, all detectors perform quite badly on the images from the visible camera at night, in comparison to FIR. What is worth highlighting is that FIR gets similar results at day and night for two of the detectors (SVM and RF) for all of the features. However, perhaps the most interesting observation is that FIR beats visible also in the daytime for all detectors. Figure 3 shows the evolution of the miss rate as FPPI increases for the cases of using HOG and HOG+LBP.

### 5.3. Experiments on KAIST Dataset

For the CVC-14 dataset, each sequence consists of a pair of video streams, one per camera, which are not perfectly synchronized. This means that, for each frame of one of the streams, say the visible one, we can always locate the closest frame in time in the other (FIR) stream, but they were not captured at the same time so they contain slight differences because camera location and orientation were not the same. Such differences are so small that the visible *vs.* FIR comparison presented here remains fair according to our purposes. However, if we want to compare the accuracy with single modality features *versus* multimodality (visible plus FIR), we need to make sure that those features correspond exactly to the same region in the scene. Fortunately, the KAIST dataset [36] was recorded with this goal in mind.

**Figure 4:**
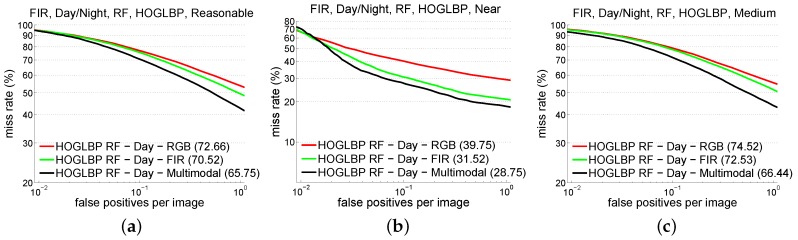
Results using different test subsets over KAIST multispectral dataset during daytime. Results obtained with RF/HOG+LBP for (**a**) reasonable (**b**) near and (**c**) medium pedestrian subsets.

From now on, we assume the use of RF/HOG+LBP, since, for the CVC-14 dataset, it was the best performing together with DPM/HOG+LBP in the daytime for visible and FIR, and it was better than DPM/HOG+LBP at nighttime using FIR (using the visible spectrum all the detectors are performing really bad). At this point, we introduce a new variation: concatenating the same features from visible and FIR. In addition, and for the sake of comparison, we run all the experiments, not only for the set of reasonable pedestrians but also to distinguish near from medium distance pedestrians.The *near* subset includes pedestrians with height equal to or higher than 75 pixels. The *medium* subset includes pedestrians between 50 and 75 pixel height. This is described in Table 4 while Figure 4 shows the complete curves when varying the number of false positives per image.

The same detector consistently gets the minimum AMR for all the cases. More significant is the fact that the best feature descriptor in the daytime results in the combination of visible and FIR features, whereas at night, FIR features achieve the maximum performance by themselves, closely followed by the combination of FIR and visible features (just about 3% more AMR on average). In this evaluation, we obtain competitive detectors in KAIST benchmark where state-of-the-art is, for daytime and reasonable pedestrians, AMR of 64.17% in the Caltech evaluation protocol, while we obtain for the same subset 65.75%.

In fact, the combination of visible + FIR at daytime is just scarcely better than using only FIR (see RF/HOG+LBP), specially for near pedestrians. Thus, from the viewpoint of the cost, it seems more reasonable to focus on improving FIR alone if the only purpose of the system is pedestrian detection. Of course, if other functionalities are required, such as traffic sign recognition in ADAS, then combining both systems is a good option as long as the synchronization of the cameras and the alignment of the images are cost effective.

**Figure 5:**
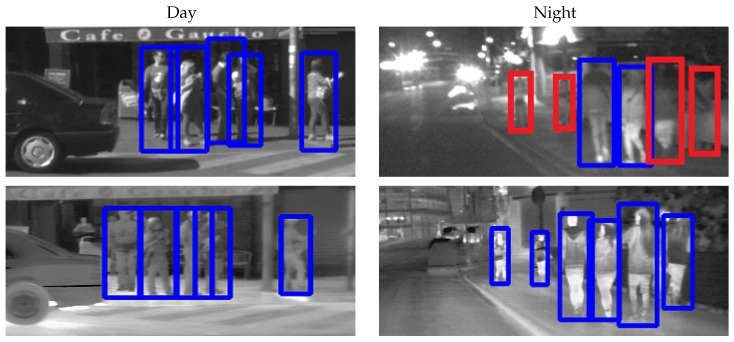
Qualitative Results comparing HOG/LinSVM detectors in different time/sensor conditions. The top row shows results over visible spectrum images, the bottom row over far infrared images. Blue boxes represent correct detections (True Positive), while red boxes represent misdetections (False Negative).

As for the CVC-14 case, visible spectrum provides very poor results at nighttime (Qualitative results in Figure 5). Our dataset has been obtained using halogen headlights and the camera is not operating at high dynamic range. Thus, it seems that, to improve nighttime results, a more sophisticated illumination system and a very well set high dynamic range scheme must be designed if we want to use such types of camera for on-board pedestrian detection.

## 6. Conclusions

In this paper, we have presented a study of pedestrian detection using commercial visible and FIR sensors operating during daytime and nighttime. This evaluation is based on well known features HOG and LBP and holistic (SVM), patch-based (RF), and part-based models (DPM), to train state-of-the-art classifiers.

The main conclusion is that the combination of features from FIR and visible images produces the best detector in the daytime by a notorious margin (about 5% less AMR) from just visible or just FIR features. This was originally unexpected since one would guess that the poor details observed in FIR images do not add discriminative power to features from visible images in the daytime. In fact, the FIR modality is as discriminative as the visible one, even not too far from the combined use of these sensors. At nighttime, FIR features get the best result and concatenating the two features’ vectors produces just a slight increase in AMR. Overall, we hope our results help to encourage the development of cheaper FIR cameras well integrated with those of the visible spectrum for developing more reliable ADAS and autonomous vehicles.

## Figures and Tables

**Table 1:** FLIR Tau 2 and UI-3240CP camera specifications.

Specifications	FLIR Tau 2	IDS UI-3240CP
Resolution	640 × 512 pixels	1280 × 1024 pixels
Pixel size	17 μm	5.3 μm
Focal length	13 mm	Adjustable (fixed 4 mm)
Sensitive area	10.88 mm × 8.7 mm	6.784 mm × 5.427 mm
Frame rate	30/25 Hz (NTSC/PAL)	60 fps

**Table 2:** New CVC-14 dataset summary of images and annotated pedestrians.

Set	Variable	FIR		Visible	
		Day	Night	Day	Night
Training	Positive Frames	2232	1386	2232	1386
	Negative Frames	1463	2004	1463	2004
	Annotated Pedestrians	2769	2222	2672	2007
	Mandatory Pedestrians	1327	1787	1514	1420
Testing	Frames	706	727	706	727
	Annotated Pedestrians	2433	1895	2302	1589
	Mandatory Pedestrians	2184	1541	2079	1333

**Table 3:** Average miss rate (AMR) in the CVC-14 dataset.

Detector		Day		Night	
		Visible	FIR	Visible	FIR
SVM	HOG	42.9	22.7	71.8	25.4
	LBP	40.6	21.6	87.6	32.1
	HOG+LBP	37.6	21.5	76.9	**22.8**
DPM	HOG	28.6	18.9	73.6	24.1
	HOG+LBP	25.2	18.3	76.4	31.6
RF	HOG	39.9	20.7	68.2	24.4
	HOG+LBP	26.6	**16.7**	81.2	24.8

**Table 4:** AMR (average miss rate) in the KAIST dataset. The three rows in each cell represent the AMR for near, medium and reasonable pedestrians, as explained in the text.

Detector		Day			Night		
		Visible	FIR	Visible + FIR	Visible	FIR	Visible + FIR
RF	HOG + LBP	39.7	31.5	**28.7**	76.0	**25.3**	29.4
		74.5	72.5	**66.4**	93.2	**60.0**	61.7
		72.7	70.5	**65.7**	91.4	**53.5**	56.7
